# Differentiation of Mountain- and Garden-Cultivated Ginseng with Different Growth Years Using HS-SPME-GC-MS Coupled with Chemometrics

**DOI:** 10.3390/molecules28052016

**Published:** 2023-02-21

**Authors:** Luoqi Zhang, Ping Wang, Sen Li, Dan Wu, Yute Zhong, Weijie Li, Haiyu Xu, Luqi Huang

**Affiliations:** 1 College of Traditional Chinese Medicine, Jilin Agricultural University, Changchun 130118, China; 2 Institute of Chinese Materia Medica, China Academy of Chinese Medical Sciences, Beijing 100700, China; 3 National Resource Center for Chinese Materia Medica, China Academy of Chinese Medical Sciences, Beijing 100700, China; 4 Experimental Research Center, China Academy of Chinese Medical Sciences, Beijing 100700, China; 5 Key Laboratory for Research and Evaluation of TCM, National Medical Products Administration, China Academy of Chinese Medical Sciences, Beijing 100700, China

**Keywords:** Mountain-Cultivated Ginseng, Garden-Cultivated Ginseng, HS-SPME-GC-MS, PCA, OPLS-DA

## Abstract

Although there are differences in the appearance of Mountain-Cultivated Ginseng (MCG) and Garden-Cultivated Ginseng (GCG), it is very difficult to distinguish them when the samples are processed to slices or powder. Moreover, there is significant price difference between them, which leads to the widespread adulteration or falsification in the market. Thus, the authentication of MCG and GCG is crucial for the effectiveness, safety, and quality stability of ginseng. In the present study, a headspace solid-phase microextraction gas chromatography mass spectrometry (HS-SPME-GC-MS) coupled with chemometrics approach was developed to characterize the volatile component profiles in MCG and GCG with 5-,10-,15-growth years, and subsequently to discover differentiating chemical markers. As a result, we characterized, for the first time, 46 volatile components from all the samples by using the NIST database and the Wiley library. The base peak intensity chromatograms were subjected to multivariate statistical analysis to comprehensively compare the chemical differences among the above samples. MCG_5-,10-,15-years_ and GCG_5-,10-,15-years_ samples were mainly divided into two groups by unsupervised principal component analysis (PCA), and 5 potential cultivation-dependent markers were discovered based on orthogonal partial least squares-discriminant analysis (OPLS-DA). Moreover, MCG_5-,10-,15-years_ samples were divided into three blocks, and 12 potential growth-year-dependent markers enabled differentiation. Similarly, GCG_5-,10-,15-years_ samples were also separated into three groups, and six potential growth-year-dependent markers were determined. The proposed approach could be applied to directly distinguish MCG and GCG with different growth years and to identify the differentiation chemo-markers, which is an important criterion for evaluating the effectiveness, safety, and quality stability of ginseng.

## 1. Introduction

Ginseng, a perennial herb of the Acanthopanax family, is known as the king of herbs and also the king of medicine, and has been used clinically for thousands of years in Asian countries. It is called “Ginseng” because its rhizome looks like a person [1]. According to the different growth environments and diverse cultivation modes, ginseng is mainly divided into three categories: Mountain-Cultivated Ginseng (MCG), Garden-Cultivated Ginseng (GCG), and Wild Ginseng (WG). MCG is planted artificially in mountain forests and grows naturally, GCG is planted artificially in farmland, while WG grows naturally in mountains and forests [2]. Different growth environments lead to the different appearance of ginseng, but it is extremely difficult to distinguish them when the ginseng samples are cut into slices or ground into powder [3]. Due to the high price and low output of WG, the market is dominated by MCG and GCG. The price of MCG is generally higher than that of GCG, so adulteration or falsification has always been widespread in the market. In addition, the different growth environment leads to the diversity of secondary metabolites, which results in different pharmacological activities and clinical values [4,5]. Thus, the differentiation of MCG and GCG is essential for the effectiveness, safety, and quality stability of ginseng.

The secondary metabolites are affected by species, growth environment and harvest time, but are not dependent on the external shape of the herbs. Therefore, sophisticated analytical techniques for the chemical phenotype are crucial for chemical composition detection. In recent years, with the development of analytical technology, ultra-high performance liquid chromatography (UHPLC) combined with mass spectrometry (MS), which has the characteristics of high sensitivity, high resolution and high precision mass measurement, has been extensively developed to discriminate ginseng based on the variety of ginsenosides [6,7,8,9]. Compared with UPLC-MS, gas chromatography-mass spectrometry (GC-MS) has a simpler sample pretreatment process and integrates sampling, concentration, and injection together, which is cost-effective and labor saving. Ginseng has its own special odor, and its aroma characteristics has attracted wide attention for ginseng identification, so GC-MS is also applied to discriminate ginseng from different habitats [10] in order to differentiate metabolites in the leaf, stem, petiole, lateral root and main root of ginseng [11], to distinguish the geographical origin of American ginseng [12], to analyze metabolic differences of ginseng berries according to cultivation age and ripening stage [13], to discriminate the three Panax species [14], and to assay the profiling in *Panax ginseng* and *Panax quinquefolius* [15].

However, GC-MS coupled with chemometrics has not been applied to systematically investigate the difference between MCG and GCG with different growth years. Therefore, in the present study, a headspace solid-phase microextraction gas chromatography mass spectrometry (HS-SPME-GC-MS) coupled with chemometrics approach was developed to discriminate the volatile component profiles in MCG_5-,10-,15-years_ and GCG_5-,10-,15-years_, and subsequently to discover differentiating chemical markers. Our study not only could systematically characterize the volatile components in ginseng, but could also provide a reliable, accurate method for distinguishing MCG and GCG samples with different growth years.

## 2. Results

### 2.1. Components Identification from MCG_5-,10-,15-years_ and GCG_5-,10-,15-years_

**Figure 1:**
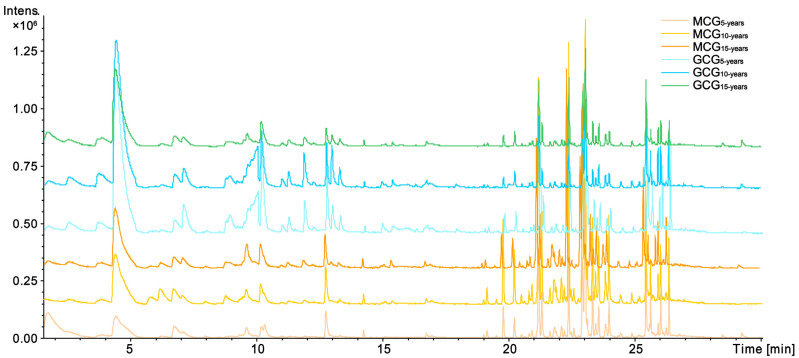
The based peak intensity (BPI) chromatograms of Garden-Cultivated Ginseng (GCG) and Mountain-Cultivated Ginseng (MCG) with 5-,10-,15-growth years by headspace solid-phase microextraction gas chromatography mass spectrometry (HS-SPME-GC-MS).

Using the optimal HS-SPME-GC-MS conditions described in Section 4.3, the representative Based Peak Intensity (BPI) chromatogram of ginseng samples is presented in Figure 1. MCG and GCG samples with different growth years have similar chemical profiles, but the difference in content of some compounds can be visually noted. Based on the NIST database and the Wiley library, a total of 46 components were preliminarily identified, including 29 sesquiterpenes, 7 carbonyl compounds, 1 pyrazine, and 9 others. The relative contents of 46 components in different samples were calculated using 2-heptanone as the internal standard (I.S.), and the detailed information is summarized in Table 1.

### 2.2. Multivariate Statistical Analysis for MCG_5–15-years_ and GCG_5–15-years_

#### 2.2.1. Principal Component Analysis (PCA)

**Figure 2:**
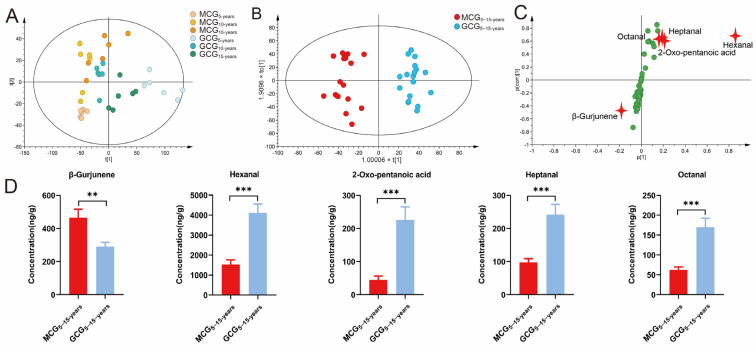
Multivariate statistical analysis base on volatile components from MCG_5–15-years_ and GCG_5–15-years_. (**A**) Principal component analysis (PCA) score plots; (**B**) Orthogonal partial least squares-discriminant analysis (OPLS-DA) score plots; (**C**) S-plots; (**D**) The contents of the five cultivation-dependent markers to distinguish MCG_5–15-years_ and GCG_5–15-years_. (*p*-value ** < 0.01; *** < 0.001).

To clearly differentiate among ginseng samples, unsupervised pattern recognition PCA, which converts multi-index data into a small number of feature components and provides visual images of large sample differences, was applied to intuitively refine group differences. After Pareto scaling and mean-centering, the dataset of MCG_5-,10-,15-years_ and GCG_5-,10-,15-years_ were displayed as score plots in a coordinate system of principal components after dimensionality reduction. As shown in Figure 2A, PCA score plots mainly separated MCG_5-,10-,15-years_ and GCG_5-,10-,15-years_ into two groups, independent of growth years, indicating that the growth environment and cultivation mode played more important roles regarding volatile secondary metabolites. Generally, R^2^X(cum) and Q2(cum) are used to evaluate the quality of mathematical models. R^2^X(cum) represents the percentage of model interpretation matrix information, Q2(cum) represents the prediction ability of the model after modeling, and both of them should be greater than 0.5 [16,17]. In the present study, R^2^X(cum) and Q2(cum) were 0.896 and 0.782, respectively, indicating good adaptability and prediction ability of the established PCA model.

#### 2.2.2. Chemo-Markers Discovery for Distinguishing MCG_5–15-years_ and GCG_5–15-years_

To determine the variables responsible for the separation between MCG_5–15-years_ and GCG_5–15-years_, the orthogonal partial least squares-discriminant analysis (OPLS-DA) approach was applied to the volatile components’ profiles. As shown in Figure 2B, OPLS-DA score plots mainly separated MCG_5–15-years_ and GCG_5–15-years_ into two blocks, especially revealing the growth-year variation in the component P1 direction (*X*-axis), and component P2 (*Y*-axis). R^2^X(cum) and Q2(cum) were 0.912 and 0.832 respectively, indicating good adaptability and prediction ability of the established OPLS-DA mode. To verify the effectiveness of the OPLS-DA model, 200 rounds of a permutation test were conducted. As shown in Figure S1A (Supplementary Materials), all blue Q2 values on the left were lower than the origin on the right, and the blue regression line of Q2 intersected the vertical axis (left) at or below zero, suggesting that our OPLS-DA model was reliable. To exhibit the responsibility of each ion for the separation more intuitively, S-plots were obtained. As shown in Figure 2C, most of the ions were gathered around the origin with only a few ions scattered around the edge area, and only the compounds represented by these few ions contributed to the separation observed in the OPLS-DA score plots. In the present study, five variables (marked in red) with Variable Importance for the Projection (VIP) > 1 and *p* < 0.05 were selected as the potential chemo-markers, which directly led to the differentiation between MCG_5–15-years_ and GCG_5–15-years_. Table 2 summarizes the detailed information of the five potential cultivation-dependent markers, including hexanal, β-gurjunene, 2-oxo-pentanoic acid, heptanal, and octanal. The contents of hexanal, 2-oxo-pentanoic acid, heptanal, and octanal in MCG_5–15-years_ were significantly higher, while β-gurjunene in GCG_5–15-years_ was significantly higher (Figure 2D). Therefore, our data suggests that the above 5 volatile components might be used as the unique chemo-markers for discrimination between MCG_5–15-years_ and GCG_5–15-years_.

### 2.3. Multivariate Statistical Analysis for MCG with 5-,10-,15-Growth Years

#### 2.3.1. PCA

**Figure 3:**
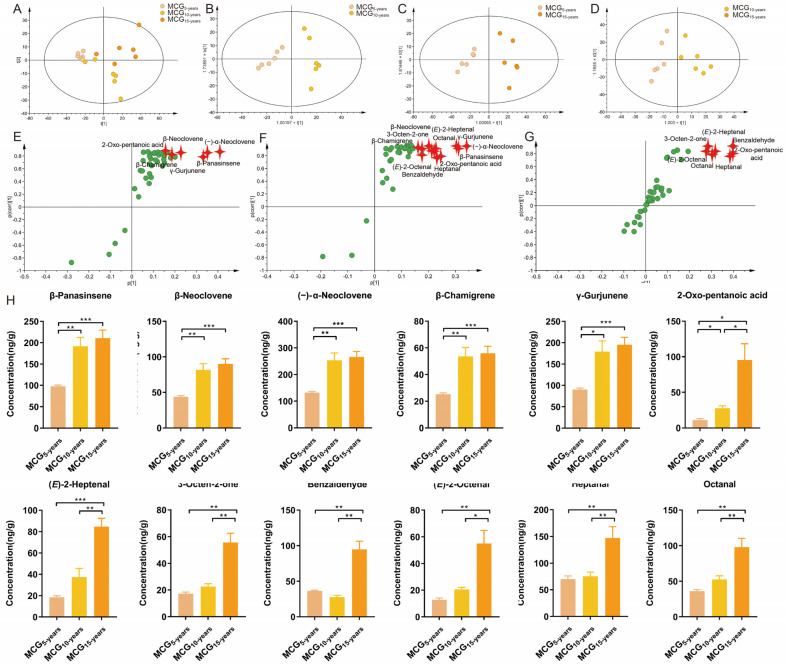
Multivariate statistical analysis based on volatile components from MCG with 5-,10-,15-growth years (**A**) PCA score plots; (**B**–**D**) OPLS-DA score plots; (**E**–**G**) S-plots; (**H**) The contents of the five growth-year-dependent markers to distinguish MCG with 5-,10-,15-growth years. (*p*-value * < 0.1; ** < 0.01; *** < 0.001).

As before, a PCA was used to intuitively refine group difference among MCG_5-,10-,15-years_. As shown in Figure 3A, the MCG_5-,10-,15-years_ were obviously separated into three blocks, indicating that the volatile component profiles of MCG change with growth years. R^2^X(cum) and Q2(cum) were 0.846 and 0.779, respectively, indicating good adaptability and prediction ability of the established PCA model (Figure 3A).

#### 2.3.2. Chemo-Markers Discovery for Distinguishing MCG with 5-, 10-, 15-Growth Years

To determine the variables responsible for the separation between MCG_5-years_ and MCG_10-years_, MCG_5-years_ and MCG_15-years_, MCG_10-years_ and MCG_15-years_, the OPLS-DA model was applied to the above dataset. As shown in Figure 3B–D, OPLS-DA score plots separated every two samples well into two blocks, with R^2^X(cum) values of 0.838, 0.9085, and 0.885, respectively, and Q2(cum) values of 0.704, 0.564, and 0.785, respectively. These models were subjected to 200 rounds of permutation tests to confirm their high predictability (Figure S1C,E,G). The ions intuitively responsible for the separation are marked in red in the S-plots (Figure 3E–G). As a result, with VIP > 1 and *p* < 0.05, six variables were determined as potential chemo-markers for discrimination between MCG_5-years_ and MCG_10-years_, seven chemo-markers were determined for the differentiation between MCG_5-years_ and MCG_15-years_, and 1 chemo-marker was determined for to distinguish between MCG_10-years_ and MCG_15-years_. Altogether, 12 potential growth-year-dependent markers, including (−)-α-neoclovene, β-panasinsene, γ-gurjunene, β-neoclovene, β-chamigrene, heptanal, 2-oxo-pentanoic acid, benzaldehyde, octanal, *(E)*-2-octenal, (*E*)-2-heptenal, and 3-octen-2-one, were determined and the information is summarized in Table 3. Of the 12 chemo-markers, except for benzaldehyde, the contents of the other 11 compounds increased with the growth years (Figure 3H), consistent with the market assessment that the higher the growth-years, the greater the value.

### 2.4. Multivariate Statistical Analysis for GCG with 5-,10-,15-Growth Years

#### 2.4.1. PCA

**Figure 4:**
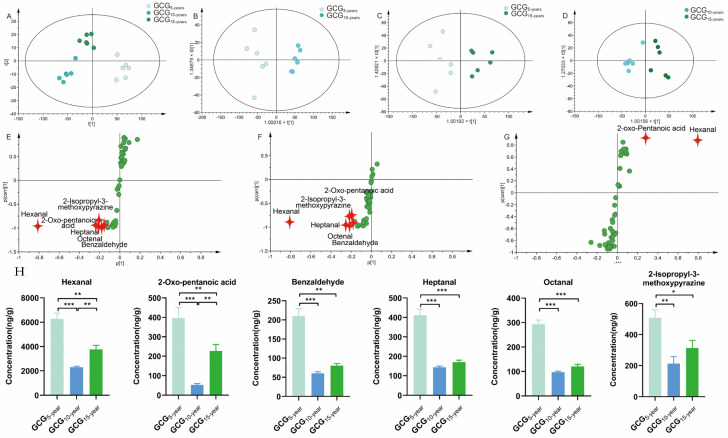
Multivariate statistical analysis base on the volatile components from GCG with 5-, 10-, 15-growth years. (**A**) PCA score plots; (**B**–**D**) OPLS-DA score plots; (**E**–**G**) S-plots; (**H**) The contents of the five growth-year-dependent markers to distinguish GCG with 5-, 10-, 15-growth years. (*p*-value * < 0.1; ** < 0.01; *** < 0.001).

Similarly, PCA was used to intuitively refine group differences among GCG with 5-, 10-, and 15-growth years. As shown in Figure 4A, PCA score plots separated GCG_5-,10-,15-years_ into three groups, indicating the chemical profile changes with the growth years, where the model fit parameters were 0.969 for R^2^X(cum), and 0.79 for Q2(cum) respectively, indicating good fitness and prediction of the established PCA model.

#### 2.4.2. Chemo-Markers Discovery for Distinguishing GCG with 5-, 10-, 15-Growth Years

To select the ions responsible for discrimination between GCG_5-years_ and GCG_10-years_, GCG_5-years_ and GCG_15-years_, GCG_10-years_ and GCG_15-years_, the above dataset was subjected to OPLS-DA. As shown in Figure 3B–D, OPLS-DA score plots significantly separated each of the two samples into two groups, where the model fit parameters were 0.938, 0.913, and 0.842 for R^2^X(cum), and 0.915, 0.728, and 0.692 for Q2(cum), respectively. These models were subjected to 200 rounds of permutation tests to confirm their high predictability (Figure S1I,K,M). The volatile components intuitively responsible for the differentiation are marked in red in the S-plots (Figure 3H–J). As a result, with VIP > 1 and *p* < 0.05, 6 ions were determined as the potential chemo-markers for discrimination between GCG_5-years_ and GCG_10-years_, six chemo-markers were determined for the differentiation between GCG_5-years_ and GCG_15-years_, and two chemo-markers were determined for the distinguishing between GCG_10-years_ and GCG_15-years_. Altogether, six potential growth-year-dependent markers, including hexanal, 2-oxo-pentanoic acid, heptanal, benzaldehyde, octanal, and 2-isopropyl-3-methoxypyrazine, were determined, and the information is summarized in Table 4. The contents of all six chemo-markers were the highest in GCG_5-years_ (Figure 4H), which is also consistent with the market assessment that GCG_5-years_ is the main commodity in the market.

## 3. Discussion

Ginsenosides are the main components in the chemical profiles of ginseng, and some prevalent analytical methods, such as UPLC-MS and ^1^H NMR are developed to distinguish different types of ginseng based on the diversity of ginsenosides. As is well known, ginseng has its own odor, and its aroma characteristics has attracted wide attention for ginseng identification. Thus, GC-MS also enables ginseng discrimination based on the volatile components.

According to their different growth environments and cultivation modes, MCG_5-,10-,15-years_ and GCG_5-,10-,15-years_ have different volatile chemical profiles. To further systematically compare the similarities and differences of the volatile components contained in MCG_5-,10-,15-years_ and GCG_5-,10-,15-years_, and particularly to compare the composition variations with growth years, HS-SPME-GC-MS coupled with chemometrics was developed to discriminate the volatile component profiles and subsequently to discover differentiating chemical markers.

First, a total of 46 volatile components were preliminarily characterized in the samples by using the NIST database and the Wiley library. According to reports in the literature, the main volatile components of ginseng are sesquiterpenoids and sesquiterpenols, which have anticancer, anti-inflammatory, and immunomodulatory pharmacological activities [18,19]. In our study, the contents of sesquiterpenes such as (−)-β-elemene, β-panaxalene, (−)-α-neobutene and γ-gultrane were high in ginseng. In addition, derivatives of methoxypyrazine, an earthy aroma component in wine, may be the main source of ginseng’s unique flavor. 2-Isobutyl-3-methoxypyrazine was detected in all groups, consistent with previous studies and, with increasing growth-years, its contents gradually increased.

Secondly, combined with multivariate analysis, it was found that different cultivation modes affected the volatile components in ginseng. Although both GCG and MCG are sources of ginseng, their cultivation modes are quite different. GCG is planted in the field, and the growth process is subject to human interference. Thus, the light exposure time, light intensity, and nutrition of GCG are much better than those of MCG. For comparison, the volatile components of *Zea mays* L. decrease significantly under light or nutrient deficiency and the contents of (*Z*)-3-hexenyl acetate and (*Z*)-3-hexen-1-ol in *Brassica napus* decreased under nitrogen deficiency [20]. This precedent indicates that light, nutrition, and temperature may be important factors for the significant differences in volatile components of ginseng, and may be the reasons why some volatile component contents were higher in GCG than in MCG [21]. However, although the contents of hexanal, 2-oxo-pentanoic acid, heptanal and octanol in GCG were higher than in MCG, it has been reported that alkane components and intermediate products such as 2-oxo-pentanoic acid contribute less to the fragrance of plants. β-Gurjunene with balsam flavor was significantly higher in MCG than that in GCG, which may be why the taste of MCG is more popular with consumers. In short, the cultivation environment leads to different volatile components among MCG and GCG. Five components were selected as potential cultivation-dependent chemo-markers for the differentiation between MCG_5-,10-,15-years_ and GCG_5-,10-,15-years_, including hexanal, 2-oxo-pentanoic acid, heptanal, octanal, and β-gurjunene. The contents of the first four compounds were significantly higher in GCG_5-,10-,15-years_, while β-gurjunene was significantly higher in MCG_5-,10-,15-years_.

Thirdly, the compositional variation with growth years was explored in MCG. A total of 12 volatile components were selected as potential growth-year-dependent chemo-markers for discrimination among MCG_5-,10-,15-years_, including β-panasinsene, β-neoclovene, (−)-α-neoclovene, β-chamigrene, γ-gurjunene, 2-oxo-pentanoic acid, (*E*)-2-heptenal, 3-octen-2-one, benzaldehyde, (*E*)-2-octenal, heptanal, and octanal. The first to sixth compounds were responsible for discrimination between MCG_5-years_ and MCG_10-,15-years_, and 2-oxo-pentanoic acid was responsible for the differentiation between MCG_10-years_ and MCG_5-,15-years_. The contents of the sixth to twelfth chemo-markers in MCG_15-years_ were significantly higher. Of the 12 chemo-markers, except for benzaldehyde, the contents of the other 11 compounds increased with the growth years, consistent with the market assessment that the higher the growth-years, the greater the value. Therefore, our data suggest that the 12 volatile components might be used as unique chemo-markers to distinguish among MCG_5-,10-,15-years_.

Finally, the compositional variation with growth years was explored in GCG. A total of six volatile components were selected as potential growth-year-dependent chemo-markers for discrimination among GCG_5-,10-,15-years_, including hexanal, 2-oxo-pentanoic acid, heptanal, benzaldehyde, octanal, and 2-isopropyl-3-methoxypyrazine. Interestingly, the contents of all six chemo-markers were the highest in GCG_5-years_, also consistent with the market assessment that GCG_5-years_ is the main commodity in the market. GCG_5-years_ is the main commodity in the market, suggesting that it has greater value than that of other growth years, which is also consistent with our results. Therefore, our data suggest that the six volatile components might be used as unique chemo-markers for discrimination among GCG_5-,10-,15-years_.

## 4. Materials and Methods

### 4.1. Chemicals and Reagents

Lazi mountain is an extension branch of Changbai Mountain, with dense forest, distinct seasons and abundant precipitation. MCG_5-,10-,15-years_ were collected from Lazi Mountain, and GCG_5-,10-,15-years_ were collected from the cultivation areas adjacent to the Lazi Mountain in Huanren County (41.26° N, 125.36° E; Benxi City, Liaoning Province, China) in September 2017. These ginseng samples were stored at −80 °C and the voucher specimens were deposited in our lab.

Methanol (Mass grade) and 2-heptone was purchased from Sigma-Aldrich (Steinheim, Germany) and NaCl from Solarbio (Beijing, China). The ultra-pure water was prepared by the Milli-Q water purification system (Millipore, Bedford, MA, USA).

### 4.2. Sample Preparation and HS-SPME-GC-MS Analysis

Ginseng samples (*n* = 6) were cut into 0.2–0.3 cm slices and dried at 37 °C for 16 h before the slices were crushed and screened through a 40-mesh sieve. A total of 100 mg of ginseng powder was transferred into a 4-mL headspace vial containing 400 µL of 20% NaCl solution that was used to disrupt the enzymatic activity of ginseng samples [22]. 2-heptone, as the internal standard, was also added into the vial with a final concentration of 0.125 ng/µL. For the volatile component analysis, a 100-µm fused silica fiber coated with DVB/PDMS/CAR was used and preheated at 40 °C for 5 min before being exposed to the headspace at 40 °C for 25 min. HS-SPME-GC-MS analysis was performed with a 7890A GC (Agilent, Palo Alto, CA, USA), equipped with a 5977B single quadrupole mass detector (Agilent, Palo Alto, CA, USA). The chromatographic separation was performed on a DB5-MS mass spectrometry column (30 m × 0.25 mm × 0.25 µm, Agilent, Palo Alto, CA, USA). The instrumental method was a modified version of Li and Gou’s work [23,24]. The injector (splitless mode) temperature was set at 270 °C. The oven temperature was initially set at 40 °C, held for 5 min, and increased to 150 °C at the rate of 5 °C/min, and then the temperature was ramped up to 260 °C at the rate of 15 °C/min and held for 7 min. The temperature of the quadruple mass analyzer was set at 150 °C. The EI ion source was used for MS data acquisition with a temperature of 230 °C and the full-scan acquisition range was from 50 to 600 amu. Helium was used as the carrier gas at a flow rate of 1 mL/min (constant flow).

### 4.3. Identification and Semi-Quantitative of Volatile Compounds

The mass spectra were used for qualitative identification of compounds by matching with the NIST mass-spectral library (NIST 11.0, National Institute of Standards and Technology, Gaithersburg, MD, USA) and the Wiley library search data system. Data analysis was performed with MassHunter qualitative (B.07.00) workstation software. Volatile compounds were semi-quantitatively analyzed using 2-heptanone as the I.S. [25,26].

$$\mathrm{concentration}\left(\frac{\mathrm{ng}}{g}\right)=\frac{\mathrm{extracted}\;\mathrm{ion}\;\mathrm{peak}\;\mathrm{area}}{\mathrm{extracted}\;\mathrm{ion}\;\mathrm{peak}\;\mathrm{area}\;\mathrm{of}\;I.S}\left[I.S.\left(\frac{10\;\mathrm{ng}}{g}\right)\right]$$

### 4.4. Statistical Analysis

SIMCA-P analysis software (version 13.0, Umetrics, Malmo, Sweden) was used for multivariate statistical analysis, including PCA and OPLS-DA. During the analysis, PCA was first used to detect clustering formation and to get the overview and classification, and OPLS-DA was then performed, aiming to determine the maximum separation between the two groups. S-plots were available to provide visualization of the OPLS-DA predictive component loading to facilitate model interpretation. VIP was used to help screen the different components. The components were screened with VIP > 1, and then a Student’s *t*-test was performed to confirm the significant difference with SPSS (SPSS 22.0; Chicago, IL, USA).

## 5. Conclusions

In summary, we characterized, for the first time, 46 volatile components in MCG_5–15-years_ and GCG_5–15-years_, and five of them were screened out to distinguish MCG_5–15-years_ and GCG_5–15-years_, 12 of them to discriminate MCG_5-,10-,15-years_, and six of them to differentiate GCG_5-,10-,15-years_. Thus, our data suggest that 15 volatile components might be used as unique chemo-markers for discrimination among MCG and GCG with different growth years. The proposed approach could be applied to directly distinguish MCG and GCG with different growth years and to identify the differentiating chemo-markers, which is an important criterion for evaluating the effectiveness, safety, and quality stability of ginseng. In addition, the research has digitized the traditional identification method of “nose smell” by HS-SPME-GC-MS, which is of great significance for the inheritance and innovation of traditional identification methods.

## Figures and Tables

**Table 1:** The contents of the volatile compounds in MCG_5-,10-,15-years_ and GCG_5-,10-,15-years_ (ng/g, *n* = 6).

No.	Compounds	MCG			GCG		
		5 Years	10 Years	15 Years	5 Years	10 Years	15 Years
1	Hexanal	876.31 ± 179.70	1160.22 ± 322.97	2551.47 ± 1154.39	6282.94 ± 1163.54	2298.02 ± 168.66	3768.41 ± 803.90
2	1-Hexanol	92.22 ± 32.58	175.40 ± 151.51	239.00 ± 180.35	145.17 ± 37.86	141.65 ± 14.87	161.44 ± 56.51
3	Heptanal	69.98 ± 14.63	75.35 ± 18.87	147.29 ± 51.59	411.09 ± 77.96	143.42 ± 14.94	170.35 ± 24.52
4	α-Pinene	20.21 ± 5.29	27.26 ± 7.75	26.40 ± 5.88	27.06 ± 22.33	24.01 ± 16.46	14.61 ± 8.86
5	Camphene	4.56 ± 1.31	6.98 ± 1.74	6.19 ± 1.01	6.94 ± 3.90	5.30 ± 3.60	3.53 ± 1.81
6	(*E*)-2-Heptenal	18.50 ± 3.32	37.36 ± 19.97	84.60 ± 19.39	144.75 ± 16.18	58.22 ± 7.25	75.36 ± 12.78
7	Benzaldehyde	36.45 ± 2.43	27.73 ± 6.52	94.85 ± 27.53	210.09 ± 48.35	60.35 ± 9.45	80.37 ± 13.16
8	β-Pinene	20.73 ± 6.04	33.19 ± 16.01	43.18 ± 49.36	63.98 ± 90.64	39.60 ± 47.11	39.70 ± 28.40
9	Octanal	36.07 ± 5.10	52.50 ± 12.61	97.95 ± 30.42	293.63 ± 41.48	96.77 ± 10.99	120.21 ± 21.23
10	(*E*)-(3,3-Dimethylcyclohexylidene)-acetaldehyde	32.58 ± 13.39	37.40 ± 16.23	28.28 ± 8.95	18.06 ± 7.82	9.71 ± 4.92	13.23 ± 5.32
11	4-Methylcyclohex-3-ene-1-carbaldehyde	2.35 ± 0.82	2.50 ± 0.79	7.36 ± 4.00	23.05 ± 3.47	9.04 ± 1.63	12.96 ± 3.12
12	3-Octen-2-one	17.09 ± 3.14	22.49 ± 5.44	55.67 ± 17.06	111.72 ± 6.21	76.01 ± 7.28	77.25 ± 9.10
13	(*E*)-2-Octenal	12.82 ± 3.23	20.49 ± 4.07	54.98 ± 24.06	153.96 ± 18.51	64.48 ± 9.80	83.46 ± 15.70
14	2-Isopropyl-3-methoxypyrazine	211.53 ± 39.65	222.50 ± 23.67	292.61 ± 43.29	509.32 ± 125.61	212.52 ± 111.73	313.61 ± 120.50
15	2-Oxo-pentanoic acid	10.96 ± 4.76	27.68 ± 8.28	95.60 ± 55.21	397.04 ± 131.61	53.36 ± 16.51	226.85 ± 237.52
16	Nonanal	11.05 ± 2.30	16.24 ± 3.05	31.98 ± 11.59	58.61 ± 13.27	24.60 ± 4.17	35.12 ± 8.22
17	(*E*)-2-Nonenal	5.93 ± 1.00	8.38 ± 1.72	17.44 ± 5.73	42.11 ± 4.04	13.47 ± 1.87	17.42 ± 3.50
18	γ-Elemene	7.36 ± 0.69	13.96 ± 4.74	11.18 ± 1.97	7.03 ± 2.88	11.91 ± 3.39	5.22 ± 1.17
19	β-Chamigrene	25.14 ± 2.57	53.46 ± 16.46	55.86 ± 12.89	34.55 ± 10.56	48.74 ± 10.46	29.71 ± 5.60
20	Cedrene-V6	18.74 ± 1.49	40.15 ± 10.86	40.73 ± 9.28	26.73 ± 7.98	34.40 ± 7.53	24.14 ± 4.52
21	β-Patchoulene	1.84 ± 0.21	4.09 ± 1.06	3.93 ± 0.94	2.73 ± 0.71	3.32 ± 0.76	2.48 ± 0.50
22	β-Maaliene	7.86 ± 0.62	16.24 ± 2.03	17.72 ± 4.10	12.29 ± 3.44	14.85 ± 3.32	10.91 ± 2.34
23	α-Gurjunene	3.95 ± 0.27	8.14 ± 2.03	8.58 ± 1.84	5.59 ± 1.62	7.25 ± 1.45	5.24 ± 1.17
24	α-Guaiene	3.11 ± 0.34	4.80 ± 1.92	5.37 ± 1.59	3.95 ± 0.74	5.10 ± 0.46	5.24 ± 1.17
25	β-Panasinsene	97.38 ± 7.43	191.78 ± 49.51	210.59 ± 46.16	143.60 ± 36.69	173.46 ± 36.88	128.88 ± 26.73
26	(−)-β-Elemene	49.56 ± 4.99	80.64 ± 23.13	71.24 ± 10.30	45.56 ± 12.11	70.57 ± 16.96	39.23 ± 9.62
27	(−)-Tricyclo[6.2.1.0(4,11)]undec-5-ene, 1,5,9,9-tetramethyl- (isocaryophyllene-I1)	10.51 ± 0.84	21.77 ± 5.75	21.76 ± 4.58	14.56 ± 3.40	18.58 ± 3.89	13.33 ± 2.90
28	(+)-Valencene	18.85 ± 1.42	40.57 ± 17.51	38.38 ± 9.89	18.53 ± 3.12	36.45 ± 6.56	15.25 ± 3.54
29	Caryophyllene	15.18 ± 1.29	35.64 ± 10.00	36.60 ± 4.97	14.07 ± 3.15	28.19 ± 5.65	15.19 ± 3.51
30	Caryophyllene-(I1)	6.71 ± 0.45	12.17 ± 3.57	14.78 ± 3.05	9.10 ± 2.55	12.93 ± 3.03	8.15 ± 1.92
31	β-Gurjunene	202.96 ± 20.50	525.85 ± 132.21	665.20 ± 89.22	206.16 ± 39.98	411.32 ± 95.02	252.53 ± 62.16
32	Eudesma-3,7(11)-diene	10.03 ± 1.17	22.86 ± 7.13	26.55 ± 4.24	9.15 ± 1.84	19.78 ± 3.90	10.29 ± 2.42
33	Decahydro-1,1,7-trimethyl-4-methylene-1H-Cycloprop[e]azulene	7.43 ± 1.13	16.12 ± 11.02	12.38 ± 4.61	4.96 ± 1.43	16.67 ± 4.19	4.11 ± 0.91
34	γ-Gurjunene	90.12 ± 7.96	178.95 ± 61.47	195.29 ± 41.95	112.51 ± 21.56	173.44 ± 29.87	104.23 ± 25.69
35	(−)-α-Neoclovene	131.56 ± 10.27	253.99 ± 65.45	265.85 ± 49.59	177.85 ± 39.88	216.23 ± 44.98	169.38 ± 36.86
36	Aromadendrene	11.73 ± 1.35	24.22 ± 10.37	25.83 ± 7.03	12.39 ± 2.06	23.75 ± 4.09	12.65 ± 3.05
37	(*Z*)-β-Farnesene	46.84 ± 3.44	72.71 ± 19.90	70.15 ± 11.89	41.40 ± 13.44	70.68 ± 17.01	36.94 ± 8.11
38	2-Isopropenyl-4a,8-dimethyl-1,2,3,4,4a,5,6,7-octahydronaphthalene	18.49 ± 1.51	32.17 ± 7.91	35.08 ± 6.24	26.67 ± 5.40	30.18 ± 6.31	23.14 ± 5.53
39	β-Neoclovene	43.79 ± 3.42	81.62 ± 21.50	90.02 ± 17.60	62.88 ± 12.28	71.98 ± 14.53	57.72 ± 13.46
40	β-Selinene	16.35 ± 1.89	35.90 ± 13.78	32.24 ± 7.25	17.45 ± 2.79	31.21 ± 5.57	17.63 ± 4.07
41	Elixene	15.09 ± 1.22	25.75 ± 8.37	29.47 ± 6.71	17.21 ± 3.12	27.22 ± 5.13	13.50 ± 3.27
42	(−)-Spathulenol	41.58 ± 5.69	51.17 ± 4.70	63.07 ± 13.45	57.36 ± 15.00	55.41 ± 5.33	44.72 ± 10.06
43	2,6,10,10-Tetramethyl-tricyclo [7.2.0.0(2,6)] undecan-5-ol	36.13 ± 5.89	26.73 ± 5.04	25.79 ± 4.67	46.72 ± 8.37	22.27 ± 6.00	18.57 ± 4.12
44	Humulene epoxide ii (−)	6.06 ± 1.13	9.09 ± 2.88	16.18 ± 4.27	19.94 ± 3.86	8.62 ± 1.87	16.93 ± 3.76
45	7(11)-Selinen-4α-ol	18.16 ± 3.27	16.98 ± 2.40	21.26 ± 3.62	27.60 ± 4.94	15.60 ± 2.03	16.35 ± 3.13
46	Ginsenol	151.00 ± 30.37	96.65 ± 12.55	100.31 ± 18.99	196.06 ± 41.56	82.16 ± 22.47	75.82 ± 18.74

**Table 2:** Detailed information of the five potential chemo-markers between MCG_5–15-years_ and GCG_5–1-5 years_.

Groups for Comparison	Q-Marker’ Name	VIP Value	*p*	MCG_5–15-years_	GCG_5–15-years_
GCG_5–15-years_ vs. MCG_5–15-years_	Hexanal	5.48586	>0.000	1529.33 ± 999.73	4116.45 ± 1860.92
	β-Gurjunene	1.43177	0.006	464.68 ± 217.47	290.00 ± 111.51
	2-Oxo-pentanoic acid	1.42528	>0.000	44.75 ± 48.39	225.75 ± 167.48
	Heptanal	1.28533	>0.000	97.54 ± 47.60	241.62 ± 131.77
	Octanal	1.11063	>0.000	62.17 ± 32.43	170.21 ± 94.00

**Table 3:** The detailed information of the five potential chemo-markers among MCG with 5-,10-,15-growth years.

Groups for Comparison	Q-Marker’ Name	VIP Value	*p*	MCG_5-years_	MCG_10-years_	MCG_15-years_
MCG_5-years_ vs. MCG_10-years_	β-Panasinsene	2.26268	0.005	97.38 ± 7.43	191.78 ± 49.51	210.59 ± 46.16
	(−)-α-Neoclovene	2.61166	0.006	131.56 ± 10.27	253.99 ± 65.45	265.85 ± 49.59
	γ-Gurjunene	2.21771	0.016	90.12 ± 7.96	178.95 ± 61.47	195.29 ± 41.95
	β-Neoclovene	1.45465	0.007	43.79 ± 3.42	81.62 ± 21.50	90.02 ± 17.60
	β-Chamigrene	1.22714	0.008	25.14 ± 2.57	53.46 ± 16.46	55.86 ± 12.89
	2-Oxo-pentanoic acid	1.03629	0.002	10.96 ± 4.76	27.68 ± 8.28	95.60 ± 55.21
MCG_5-years_ vs. MCG_15-year_	(−)-α-Neoclovene	2.16242	>0.000	131.56 ± 10.27	253.99 ± 65.45	265.85 ± 49.59
	β-Panasinsene	1.99593	>0.000	97.38 ± 7.43	191.78 ± 49.51	210.59 ± 46.16
	γ-Gurjunene	1.89388	>0.000	90.12 ± 7.96	178.95 ± 61.47	195.29 ± 41.95
	2-Oxo-pentanoic acid	1.76019	0.013	10.96 ± 4.76	27.68 ± 8.28	95.60 ± 55.21
	Heptanal	1.67883	0.005	69.98 ± 14.63	75.35 ± 18.87	147.29 ± 51.59
	Octanal	1.50643	0.004	36.07 ± 5.10	52.50 ± 12.61	97.95 ± 30.42
	Benzaldehyde	1.45653	0.003	36.45 ± 2.43	27.73 ± 6.52	94.85 ± 27.53
	(*E*)-2-Heptenal	1.39969	0.000	18.50 ± 3.32	37.36 ± 19.97	84.60 ± 19.39
	β-Neoclovene	1.27773	>0.000	43.79 ± 3.42	81.62 ± 21.50	90.02 ± 17.60
	(*E*)-2-Octenal	1.24425	0.007	12.82 ± 3.23	20.49 ± 4.07	54.98 ± 24.06
	3-Octen-2-one	1.18196	0.002	17.09 ± 3.14	22.49 ± 5.44	55.67 ± 17.06
	β-Chamigrene	1.03703	>0.000	25.14 ± 2.57	53.46 ± 16.46	55.86 ± 12.89
MCG_10-years_ vs. MCG_15-years_	Heptanal	2.46311	0.009	69.98 ± 14.63	75.35 ± 18.87	147.29 ± 51.59
	2-Oxo-pentanoic acid	2.42858	0.029	10.96 ± 4.76	27.68 ± 8.28	95.60 ± 55.21
	Benzaldehyde	2.13574	0.001	36.45 ± 2.43	27.73 ± 6.52	94.85 ± 27.53
	Octanal	1.96282	0.007	36.07 ± 5.10	52.50 ± 12.61	97.95 ± 30.42
	(*E*)-2-Octenal	1.70095	0.016	12.82 ± 3.23	20.49 ± 4.07	54.98 ± 24.06
	(*E*)-2-Heptenal	1.6901	0.002	18.50 ± 3.32	37.36 ± 19.97	84.60 ± 19.39
	3-Octen-2-one	1.56645	0.004	17.09 ± 3.14	22.49 ± 5.44	55.67 ± 17.06

**Table 4:** The detailed information of the six potential chemo-markers among GCG with 5-,10-,15-growth years.

Groups for Comparison	Q-Marker’ Name	VIP Value	*p*	GCG_5-years_	GCG_10-years_	GCG_15-years_
GCG_5-years_ vs. GCG_10-years_	Hexanal	5.45061	>0.000	6282.94 ± 1163.54	2298.02 ± 168.66	3768.41 ± 803.90
	2-Oxo-pentanoic acid	1.60289	>0.000	397.04 ± 131.61	53.36 ± 16.51	226.85 ± 237.52
	Heptanal	1.40708	>0.000	411.09 ± 77.96	143.42 ± 14.94	170.35 ± 24.52
	Benzaldehyde	1.03157	>0.000	210.09 ± 48.35	60.35 ± 9.45	80.37 ± 13.16
	Octanal	1.20191	>0.000	293.63 ± 41.48	96.77 ± 10.99	120.21 ± 21.23
	2-Isopropyl-3-methoxypyrazine	1.46396	0.001	509.32 ± 125.61	212.52 ± 111.73	313.61 ± 120.50
GCG_5-years_ vs. GCG_15-years_	Hexanal	5.41556	0.001	6282.94 ± 1163.54	2298.02 ± 168.66	3768.41 ± 803.90
	2-Oxo-pentanoic acid	1.44353	0.023	397.04 ± 131.61	53.36 ± 16.51	226.85 ± 237.52
	Heptanal	1.61564	>0.000	411.09 ± 77.96	143.42 ± 14.94	170.35 ± 24.52
	Benzaldehyde	1.13916	0.001	210.09 ± 48.35	60.35 ± 9.45	80.37 ± 13.16
	Octanal	1.36848	>0.000	293.63 ± 41.48	96.77 ± 10.99	120.21 ± 21.23
	2-Isopropyl-3-methoxypyrazine	1.52673	0.020	509.32 ± 125.61	212.52 ± 111.73	313.61 ± 120.50
GCG_10-years_ vs. GCG_15-years_	Hexanal	5.33736	0.006	6282.94 ± 1163.54	2298.02 ± 168.66	3768.41 ± 803.90
	2-Oxo-pentanoic acid	1.82677	0.003	397.04 ± 131.61	53.36 ± 16.51	226.85 ± 237.52

## Data Availability

The data presented in this study are available in Supplementary Materials.

## Supplementary Materials

The following supporting information can be downloaded at: https://www.mdpi.com/article/10.3390/molecules28052016/s1, Figure S1: 200 permutation tests and VIP plots for the OPLS-DA models. MCG_5-15 years_ VS GCG_5-15 years_ (A,B); MCG_5-years_ VS MCG_10-years_ (C,D); MCG_5-years_ VS MCG_15-years_ (E,F); MCG_10-years_ VS MCG_15-years_ (G,H); GCG_5-years_ VS GCG_10-years_ (I,J); GCG_5-years_ VS GCG_15-years_ (K,L); GCG_10-years_ VS GCG_15-years_ (M,N).

## Sample Availability

Samples of the compounds are available from the authors.

## Abbreviations

BPI	Based Peak Intensity
GCG	Garden-Cultivated Ginseng
GC-MS	gas chromatography-mass spectrometry
HS-SPME-GC-MS	headspace solid-phase microextraction gas chromatography mass spectrometry
I.S.	Internal standard
MCG	Mountain-Cultivated Ginseng
MS	mass spectrometry
OPLS-DA	orthogonal partial least squares-discriminant analysis
PCA	principal component analysis
UHPLC	ultra-high performance liquid chromatograph
VIP	Variable importance for the projection
WG	Wild Ginseng
