# Supplementary material for: Differentiation of Mountain- and Garden-Cultivated Ginseng with Different Growth Years Using HS-SPME-GC-MS Coupled with Chemometrics
Source: Molecules. 2023 Feb 21;28(5):2016. doi: 10.3390/molecules28052016 (PMC10004156; doi:10.3390/molecules28052016)
Supplement: Supplementary file 1 [file molecules-28-02016-s001.zip › molecules-2159400-supplementary.pdf]

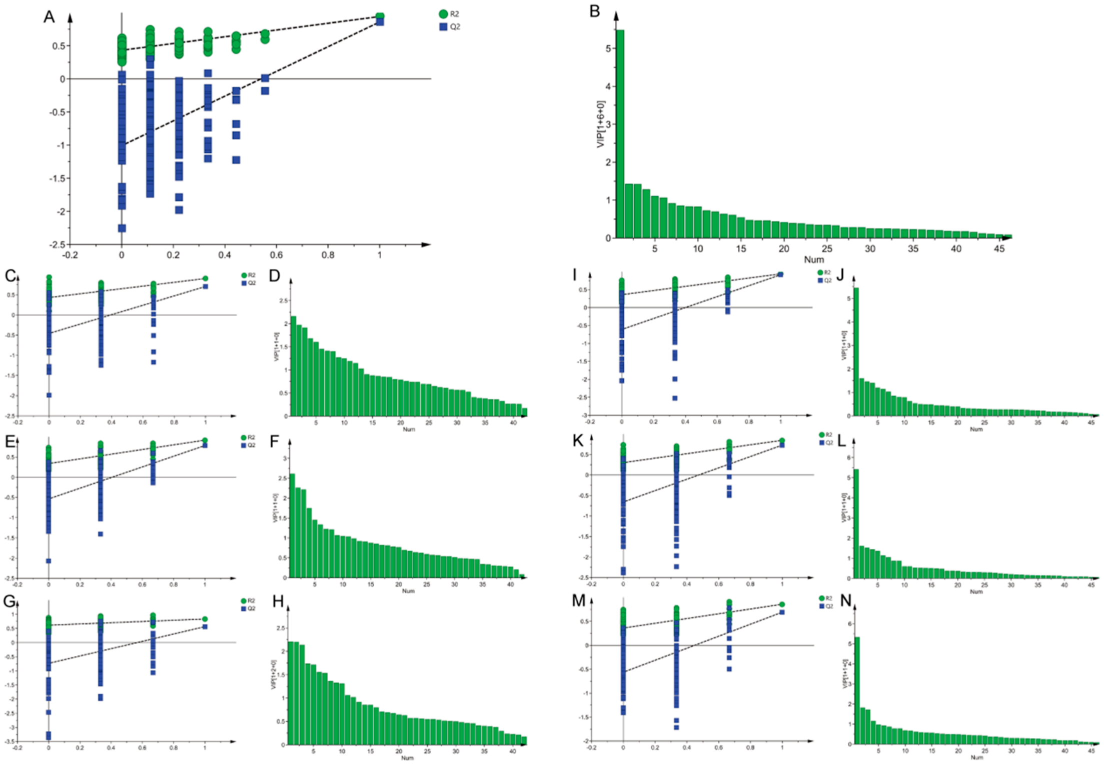

Figure S1. 200 permutation tests and VIP plots for the OPLS-DA models. MCG$_{5\text{-}15\ years}$ VS GCG$_{5\text{-}15\ years}$ (A, B); MCG$_{5\text{-}years}$ VS MCG$_{10\text{-}years}$ (C, D); MCG$_{5\text{-}years}$ VS MCG$_{15\text{-}years}$ (E, F); MCG$_{10\text{-}years}$ VS MCG$_{15\text{-}years}$ (G, H); GCG$_{5\text{-}years}$ VS GCG$_{10\text{-}years}$ (I, J); GCG$_{5\text{-}years}$ VS GCG$_{15\text{-}years}$ (K, L); GCG$_{10\text{-}years}$ VS GCG$_{15\text{-}years}$ (M, N).
